# *AdmixSim 2*: a forward-time simulator for modeling complex population admixture

**DOI:** 10.1186/s12859-021-04415-x

**Published:** 2021-10-18

**Authors:** Rui Zhang, Chang Liu, Kai Yuan, Xumin Ni, Yuwen Pan, Shuhua Xu

**Affiliations:** 1 Key Laboratory of Computational Biology, Shanghai Institute of Nutrition and Health, University of Chinese Academy of Sciences, Chinese Academy of Sciences, Shanghai, 200031 China; 2 Department of Mathematics, School of Science, Beijing Jiaotong University, Beijing, 100044 China; 3 State Key Laboratory of Genetic Engineering, Collaborative Innovation Center of Genetics and Development, School of Life Sciences, Fudan University, Shanghai, 200438 China; 4 Human Phenome Institute, Fudan University, Shanghai, 201203 China; 5 School of Life Science and Technology, ShanghaiTech University, Shanghai, 201210 China; 6 Center for Excellence in Animal Evolution and Genetics, Chinese Academy of Sciences, Kunming, 650223 China; 7 Henan Institute of Medical and Pharmaceutical Sciences, Zhengzhou University, Zhengzhou, 450052 China

**Keywords:** Population admixture, Forward-time simulation, Admixture models, Multiple-wave admixture, Evolutionary forces

## Abstract

**Background:**

Computer simulations have been widely applied in population genetics and evolutionary studies. A great deal of effort has been made over the past two decades in developing simulation tools. However, there are not many simulation tools suitable for studying population admixture.

**Results:**

We here developed a forward-time simulator, *AdmixSim 2*, an individual-based tool that can flexibly and efficiently simulate population genomics data under complex evolutionary scenarios. Unlike its previous version, *AdmixSim 2* is based on the extended Wright-Fisher model, and it implements many common evolutionary parameters to involve gene flow, natural selection, recombination, and mutation, which allow users to freely design and simulate any complex scenario involving population admixture. *AdmixSim 2* can be used to simulate data of dioecious or monoecious populations, autosomes, or sex chromosomes. To our best knowledge, there are no similar tools available for the purpose of simulation of complex population admixture. Using empirical or previously simulated genomic data as input, *AdmixSim 2* provides phased haplotype data for the convenience of further admixture-related analyses such as local ancestry inference, association studies, and other applications. We here evaluate the performance of *AdmixSim 2* based on simulated data and validated functions via comparative analysis of simulated data and empirical data of African American, Mexican, and Uyghur populations.

**Conclusions:**

*AdmixSim 2* is a flexible simulation tool expected to facilitate the study of complex population admixture in various situations.

**Supplementary Information:**

The online version contains supplementary material available at 10.1186/s12859-021-04415-x.

## Background

Computer simulation has come to play an increasingly critical role in various population genetic studies, such as estimating summary statistics of sequencing-level data, evaluating the robustness of mathematical inference frameworks, and understanding current genetic diversity underlying different demographic histories. A number of simulation tools have been developed, and some reviews have comprehensively covered the works in which they were used [1–6]. The available simulation tools can be divided into two distinct classes: backward-time, also called coalescent-based, and forward-time. Backward-time simulation tools are based on the idea of tracing back to the most recent common ancestor of currently surviving individuals and then attributing genetic information to each individual on the coalescent tree. These tools are very efficient and memory-saving because only existing individuals are considered. However, they are not powerful in complex demographic scenarios. Coalescent-based methods are not suitable for cases involving questions of interest that require complete ancestral information since only the ancestral information of currently surviving individuals is saved. Unlike coalescent-based methods, forward-time approaches start from some initial reference populations and then implement specific evolutionary events generation by generation, such as recombination, de novo mutation, natural selection, and migration. Many efficient forward-time tools have been developed recently. The National Cancer Institute’s Genetic Simulation Resources (GSR) website (https://surveillance.cancer.gov/genetic-simulation-resources/packages/) listed 178 simulators. These tools were initially designed for various modeling emphases and objectives, so that they have been widely applied to different genetics studies, such as the human epidemiology (*simuPOP* [7, 8]), the ecology of wild species (*Nemo* [9]), and the genome-wide association studies (*genomeSIM* [10], *genomeSIMLA* [11]). Among forward-time simulators, some possess functions to simulate a process of population admixture directly, such as *admix’em* [12], *SELAM* [13], *SLiM* [14–16], *simuPOP*, *fwdpp* [17], and *forqs* [18]. They are flexible and computationally efficient in fields of their respective designing objectives and also workable on other demographic scenarios. In particular, *admix’em* focuses simulations on two-way admixture. *SELAM* is especially useful in simulating admixture resulted from a founding population with all subpopulations being co-existing throughout the simulation. *SLiM* and *forqs* are both powerful in simulating the evolution of quantitative traits when assuming various demographic histories. *simuPOP* and *fdwpp* are especially suitable for users who are good at Python or Cpp programming. In addition, *SLiM 3.3* [16], *GeneEvolve* [19], *simuPOP*, and *AdmixSim* [20] also support real genomic data as input, this feature is helpful in many applications, especially when mutation-drift equilibrium is to be approached which can be time-consuming. Taken together, there is room for developing tools specifically for modeling complex population admixture. Here, we introduce a highly flexible and user-friendly forward-time simulation tool called *AdmixSim 2*. It allows simulating real sequence data in a series of complex admixture processes with various evolutionary driving forces including de novo mutation, drift, recombination, and natural selection. Notably, *AdmixSim 2* can also be applied in admixture scenarios for sex chromosomes and non-human species. The source code of *AdmixSim 2* is freely available at https://www.picb.ac.cn/PGG/resource.php or https://github.com/Shuhua-Group/AdmixSim2.

## Implementation

The data structure of *AdmixSim 2* can be mainly divided into five layers: segment, chromosome, individual, single-generation population, and multiple-generation population. *AdmixSim 2* begins with a set of individuals from ancestral populations, corresponding sex settings, chromosome information, and a user-defined demographic model. Each Individual is a diploid and constituted by two chromosomes. In the founding generation, each chromosome of ancestral individuals is assigned with a unique integer-type label ranging from 0 to $2N - 1\left( {N = \mathop \sum \limits_{i = 1}^{k} N_{i} } \right)$, $N_{i}$ is the number of individuals of the *i*th ancestral population, *i* = 1, 2, …, *k*). Chromosomes of a certain population take consecutive labels. Following the demographic model settings, for each population, the probability of being sampled to be a parent of newly generated individuals is proportional to the corresponding admixture proportion. More specifically, when there are three ancestral populations, i.e., pop1, pop2, and pop3, their admixture proportions are 0.2, 0.1, and 0.7 respectively. To make new individual data, we first generate a random number between 0 and 1. One individual of pop1 is selected if the random number is less than 0.2, while one individual of pop2 is selected if the random number is larger than 0.2 and less than 0.3, otherwise, one individual of pop3 is selected. Within each population, the probability of being sampled for each individual is proportional to the corresponding fitness. Each chromosome of newly generated individuals consists of a list of segments. Each segment is identified by the start physical position, the end physical position, and the label it originates from. The new generated individual inherits corresponding segments from parents with additional segments resulted from recombination. Asides from the ancestral segments, each chromosome has two additional lists recording the recombination breakpoints and de novo mutation points, respectively, and one map recording the physical positions under selection and corresponding states of the allele. The generation of new recombination and mutation points can be modeled as the Poisson process and assigned along the chromosome based on the user-defined recombination rate and mutation rate, respectively. Each population in *AdmixSim 2* is constituted by individuals spreading several generations specified in the demographic model.

**Fig. 1:**
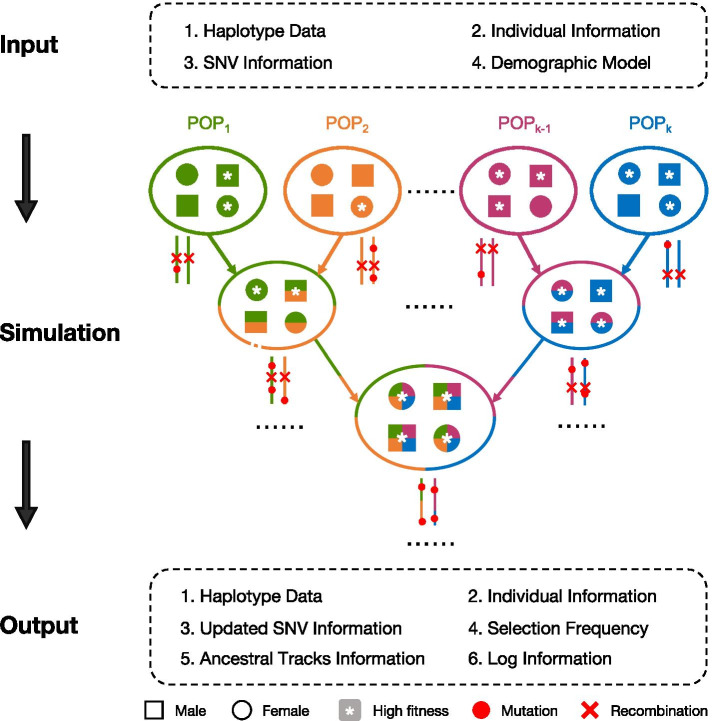
General simulation flow chart of *AdmixSim 2*. Colors indicate different ancestral populations, squares represent males, circles represent females. Red spots represent mutations and red crosses represent recombinations. *AdmixSim2* requires four input files separately recording ancestral haplotype data, individual information, SNV information, and demographic model. During the simulation, each individual in the current generation undergoes mutation and is then sampled to be a parent of offspring in the next generation. The probability of being included in this sample is proportional to the individual’s fitness. Each parent contributes one gamete to the offspring after recombination. At the end of the simulation, there are six output files. The first three take nearly the same format as the corresponding input, and they can be used for subsequent simulations with a new demographic model

Next, we provide a brief description of the workflow of *AdmixSim 2*. Detailed information can be found in the user’s manual. *AdmixSim 2* follows the extended Wright-Fisher (WF) model, which includes diploid individuals and non-overlapping, discrete generations. Unlike the strict WF model, the population size over generations can fluctuate and can be defined by users. Figure 1 illustrates the general flow chart of *AdmixSim 2*. Briefly, four input files are required for ancestral haplotype data, individual information, single nucleotide variation (SNV) information, and model description. The model description file defines the overall simulation processes by combining a series of modules of a standard format for every single process. Even though it is possible to use models in combination for extensive admixture scenarios, modifications of a single model are also applicable and flexible. With the input of these four files, *AdmixSim 2* implements the whole simulation process defined in the model description file across generations. At the end of the simulation, haplotype data of individuals of specified populations are provided, and users can flexibly sample a certain number of individual data for output. Output data of any generation are available for all of the simulated populations. The output individual information file, haplotype data file, and updated SNV information file are in a similar format as the input file, which provides convenience and flexibility for subsequent simulations across different demographic models. Moreover, the haplotype data as provided by *AdmixSim 2* are helpful for follow-up analysis and revealing the properties of real genomic data, which are not available in many other simulation tools. In addition to the aforementioned output files, *AdmixSim 2* also generates three additional outputs, frequency of alleles under selection, ancestral track information, and a log file.

Admixture, de novo mutation, and natural selection are the evolutionary events that we mainly considered in *AdmixSim 2*. Corresponding parameters can be set in input files or using the command-line interface. Initial settings for sex in the individual information file define monoecious and dioecious individuals for simulation. In the case of monoecious individuals, we logically sample two different individuals as parents of each offspring in the next generation. The probability that samples an individual out is proportional to the individual’s fitness. The fitness of an individual is calculated as one plus the sum of selection coefficients of all selection conditions satisfied. The negative value is rescaled to zero. Each parent contributes one gamete to the offspring with mutations and recombination. The recombination rate and mutation rate can be set as uniform or nonuniform (locus-specific) in either the command line or the SNV information file. For selection, *AdmixSim 2* can simulate the case of a single locus, multi-locus (continuous or discrete), population-specific, and sex-specific selection. Here, the sex-specific selection is incorporated by assigning different selection coefficients for two sexes. Sex-specific population sizes and sex-specific admixture proportions are supported as well. The selection coefficients are allowed to change over generations. Users can also simplify the simulation by using a single parameter setting to avoid complex evolutionary processes with recombination, de novo mutation, and natural selection, respectively.

We implemented some features for the efficiency of run time and memory usage. First of all, we record the recombination segments rather than the whole haplotype data, which is much more memory-efficient as stated in *forqs*. The time consuming is insensitive to the number of simulated SNVs and remains relatively small even with a large recombination rate and mutation rate. Second, we discard the population information of the previous generation after simulating the current generation unless it is specified to be output. Generally speaking, there are data of two generations existing simultaneously in the simulation process. Finally, we record the haplotype data of ancestral populations into memory after simulating all the generations instead of at the beginning. The ancestral haplotype data are used to extract the segmental sequence based on the segment information carried by each output haplotype. We compared the basic features and abilities of *AdmixSim 2* with *QuantiNemo 2* [21], *SLiM 3.3*, *simuPOP*, *GeneEvolve*, *SELAM*, *forqs*, and *admix’em* (Table 1). The results showed that *AdmixSim 2* is an irreplaceable tool for simulating population admixture with real genomic data.

**Table 1:** Comparison of the features

Simulator features	***AdmixSim 2***	***quantiNemo 2***	***SLiM 3.3***	***simuPOP***	***GeneEvolve***	***SELAM***	***forqs***	***Admix’em***
Admixture^a^	Y, SS	N	Y	Y, SS	N	Y	Y	Y
Real sequence data input	Y	N	Y/N	Y/N	Y	N	N	N
Genomic data output	Y	Y	Y	Y	Y	N	Y	N
Recombination	U, NU, A	U, NU, SS	U, NU	U, NU	U, NU	U	U, NU	U, NU, SS
Mutation	U, NU, A	U, NU, KA, SM	U, NU, KA, SM	U, NU, KA, SM	U, NU, A	U	U, NU, A	U, NU
Selection	SL, ML, POS, SS, GS, DS, A	SL, ML, POS, DS, GS, BS, ES	SL, ML, POS, DS, BS, ES, PHS, A	SL, ML, POS, BS, DS, ES	SL, ML, POS, DS, BS, PHS, A	SL, SS, POS, DS, ES	SL, ML, DS, A	SL, ML, POS, SS, DS, GS, ES, PHS
X chromosome	Y	Y	Y	Y	N	Y	Y	Y
Programming language	C++, Python	C++	C++	C++	C++	C++	C++	C++
Interface	Command-line, configuration files	Command line, configuration files	Edios programming	Python programming	Command-line, configuration files	Command-line, configuration files	Command-line, configuration files	Command-line, configuration files

^a^Generate new admixed populations

*A* Absent, *BS* Balancing Selection, *ES* Epistatic Selection, *DS* Directional Selection, *GS* Generation Specific, *KA* k-Allele Model, *ML* Multi-locus, *N* No, *NU* Nonuniform, *PHS* Phenotype Specific, *POS* Population Specific, *SL* Single-locus, *SM* Stepwise Mutation Model, *SS* Sex Specific, *U* Uniform, *Y* Yes

## Results

### Admixture, recombination, and mutation

**Fig. 2:**
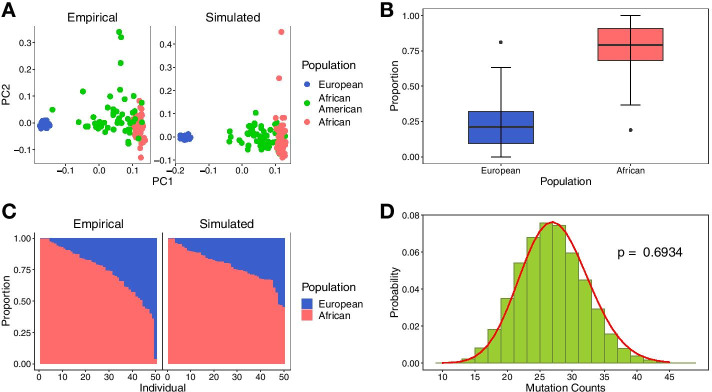
Simulation of African American admixture pattern. **A** PCA results. The left is the result of empirical data and the right is the result of simulated data. The patterns of these two results are quite similar. **B** Segment length proportion. The proportion of each ancestry was calculated based on the sum of the corresponding segment length. The average proportions of European and African were 0.225 and 0.775, which is in broad agreement with proportions set in the admixture model (European: 0.246, African: 0.754). **C** Supervised admixture analysis results at K = 2. The left is the result of empirical data and the right is simulated data. There is no marked difference between these two results. **D** Mutation number counts. The green histogram represents the simulation value and the red curve is derived from the theoretical Poisson distribution. The *p*-value was calculated using the chi-square goodness of fit test. The chromosome length was approximate 2.49 Morgan and the mutation rate was set as 10^–8^ per generation per site. Thus, after simulating 11 generations, the average mutation number of each haplotype is about 27

To validate the basic functions of our simulator, we used admixture models of three typical admixed populations (African Americans, Mexicans, and Uyghurs) which are of 2, 3, and 4 distinct ancestral originations, respectively (Additional file 4: Table S1). The simulation model of each population is shown in Additional file 1: Figure S1 [22, 23]. To simulate African Americans, we chose populations European and African from the 1000 Genomes Project (KGP) as reference. Totally 6,196,135 SNVs on chromosome 1 were used for simulation. We performed principal component analysis (PCA) with *flashpca* [24] (Fig. 2A) and supervised admixture analysis with *ADMIXTURE* [25] (Fig. 2C) of both simulated and empirical data. The results of PCA and admixture analysis were largely concordant between simulated data and empirical data, indicating the simulated data by *AdmixSim 2* are a satisfactory alternative on a global level. We further calculated the segment length proportion of each ancestral population to validate the function of sampling patterns and recombination. The variation of these proportions was largely stochastic without significant deviation from the expected value as set in the admixture model (Fig. 2B). To validate the function of adding mutations, we set the mutation rate as 10^–8^ per generation per site and compared the distribution of mutation counts of each output haplotype to the Poisson distribution (Fig. 2D). The results show that the two distributions were extremely close (*p* = 0.69*,* chi-square goodness of fit test). These results further demonstrated the simulated data by *AdmixSim 2* are a satisfactory alternative on the level of local genomic regions. For the other two admixed populations, Mexicans and Uyghurs, the aforementioned features of simulated data were confirmed (Additional file 2: Figure S2 and Additional file 3: Figure S3).

### Recombination and length distribution of segments of distinct ancestry in the analysis of X chromosome and autosomal data

**Fig. 3:**
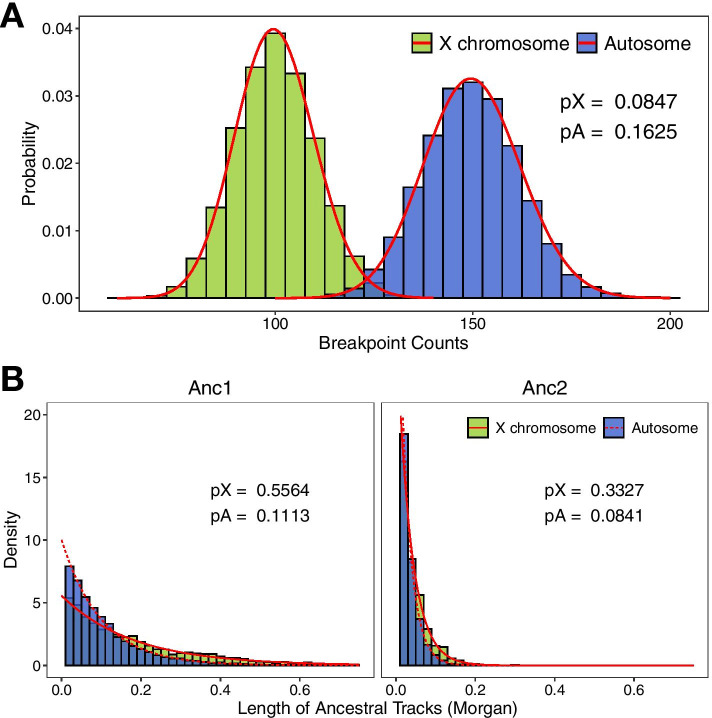
Recombination and length distribution of segments of distinct ancestry in the analysis of X chromosome and autosomal data. **A** Recombination breakpoint counts. The histogram represents the simulated value of recombination breakpoints and the red curve is the theoretical Poisson distribution. Under identical simulation models and parameter settings, the average number of recombination breakpoints of the X chromosome is approximately two-thirds of that of autosomes. **B** Segment length distribution. The histogram represents the simulated segment length distribution and the red curve is the theoretical exponential distribution. The theoretical distribution fits quite well with the simulated one. Moreover, X chromosomes possess a smaller effective recombination rate compared to autosomes

The main difference is the recombination pattern in simulating X chromosome and autosome data. While simulating X chromosome data, we assume that recombination only occurs in females. Thus, the number of recombination events of the X chromosome is expected to be two-thirds of that of autosome theoretically. We simulated an admixed population with a population size of 5000 using pseudo data of 3 Morgan. The simulation was based on hybrid isolation (HI) model with the initial ancestral contribution of 4:1 for two ancestral populations (hereafter refers to Anc1 and Anc2, respectively) and ended at generation 50. The recombination rate was set as 10^–8^ Morgan per base pair. There are differences between the numbers of recombination breakpoints of autosomes and those of X chromosome under the identical simulation model and parameter settings (Fig. 3A). The mean numbers of breakpoints of autosome and X chromosome were 149.66 and 100, respectively, thus satisfied the two-thirds ratio as expected. Next, we examined the consistency between length distribution of ancestral segments in simulation and that in the theoretical deduction based on the method proposed in *MultiWaver* [22]. The statistical significance was determined by the Kolmogorov–Smirnov test. As expected, the two distributions did not show statistical significance, which again demonstrated a fundamental capacity of *AdmixSim 2* to simulate X chromosome data (Fig. 3B).

### Natural selection

To verify the frequency variation tendency of alleles under selection, we simulated a two-locus segment under a selection amenable to the additive model with an initial freqncy of 5% and a selection coefficient of 0.1. Under the additive model, the fitness is 1, 1 + *s*, and 1 + 2* s* for individuals carrying 0, 1, and 2 selected haplotypes, respectively, where *s* denotes the selection coefficient. During each simulation of 500 repeats, we recorded the selection frequency at each generation. Using *x*_*t*_ to denote allele frequency at generation *t*, we have the theoretical frequency at generation *t* + 1 as

$$x_{t + 1} = \frac{{\left( {1 + 2s} \right)x_{t}^{2} + x_{t} \left( {1 - x_{t} } \right)\left( {1 + s} \right)}}{{\left( {1 + 2s} \right)x_{t}^{2} + 2x_{t} \left( {1 - x_{t} } \right)\left( {1 + s} \right) + (1 - x_{t} )^{2} }}.$$

**Fig. 4:**
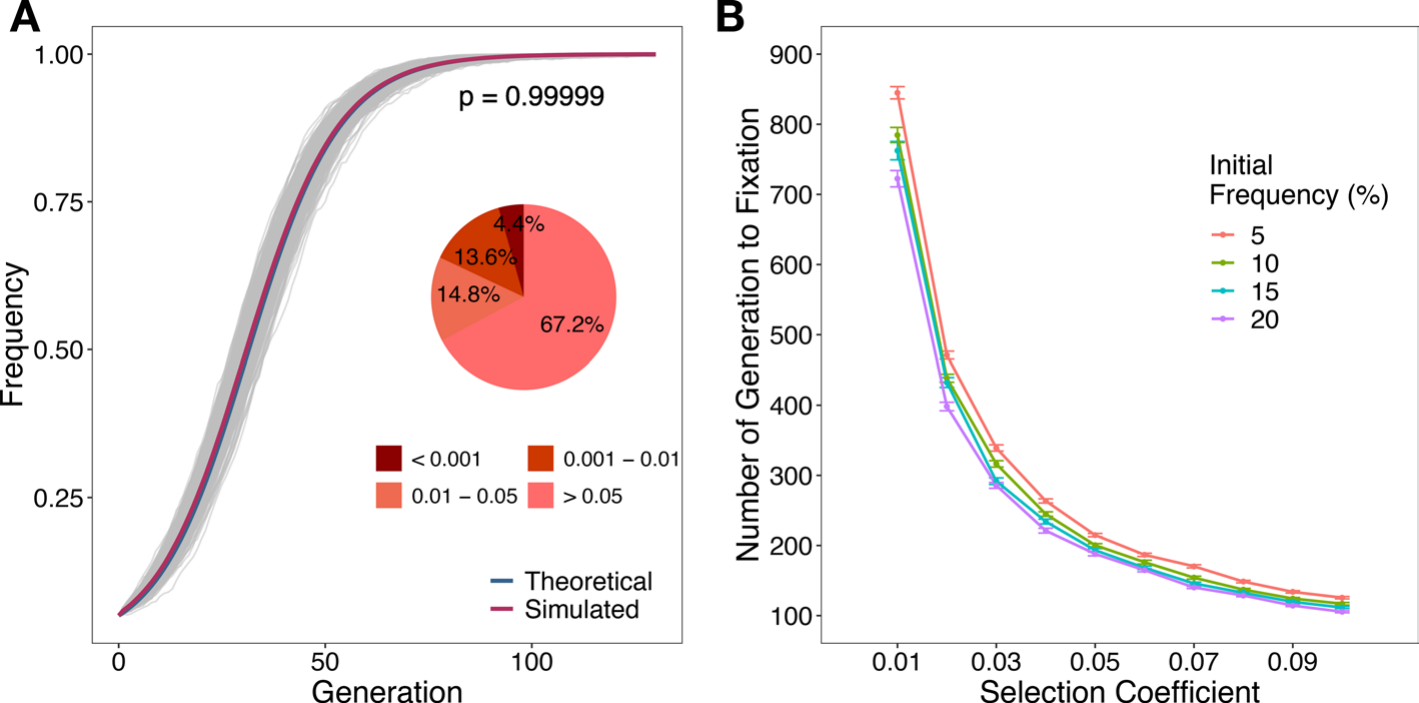
Selection Test of *AdmixSim 2*. **A** The variation trend of allele frequency. The grey curve represents each repeat. The red curve is the average of 500 repeats and the blue one is the theoretical value. The *p*-value was calculated using the Kolmogorov–Smirnov test and the average of 500 simulations was quite indistinguishable from the theoretical one. The pie chart on the right shows the statistical analysis of each repeat and the theoretical value. More than 95% of tests did not show statistical significance. **B** Allele fixation time under different combinations of initial frequency and selection coefficient. The fixation time reasonably decreased with the increase of selection coefficients. Moreover, the higher initial frequencies were, the fewer generations were cost to reach a fixation state

Given the condition of $x_{0} = 0.05$ and $s = 0.1$, the mean value of allele frequencies across 500 repeats was compared to the expected value at each generation (Fig. 4A). The results of simulation and theoretical expectation are consistent regarding the frequency of allele under selection (*p* = 0.999*,* Kolmogorov–Smirnov test).

To test the number of generations of an allele being fixed under selections with different initial frequencies and coefficients, we simulated 1 centiMorgan chromosome and used the HI model with an initial contribution of 1:1 from two ancestral populations. Each ancestral population had 100 individuals in the founding generation and the population size of the admixed population was 5,000 throughout the simulation. Recombination rate (Morgan per base pair) and mutation rate (per generation per site) were both set as 10^–8^. We considered four distinct initial frequencies of the segment under selection, 5%, 10%, 15%, and 20%. The selection coefficient varied in the change of 0.01–0.1, taking 0.01 as a step (Additional file 5: Table S2). For each combination of initial frequency and selection coefficient setting, we performed 100 replications, and then calculated the average of fixation time with standard error (Fig. 4B). As expected, the fixation time decreased with the increase of selection coefficients. Besides, there was a negative correlation between the initial frequencies and the time in generations taken to reach a fixation state. These results revealed a powerful performance of *AdmixSim 2* in terms of simulating natural selection during admixture.

### Time and memory cost

**Fig. 5:**
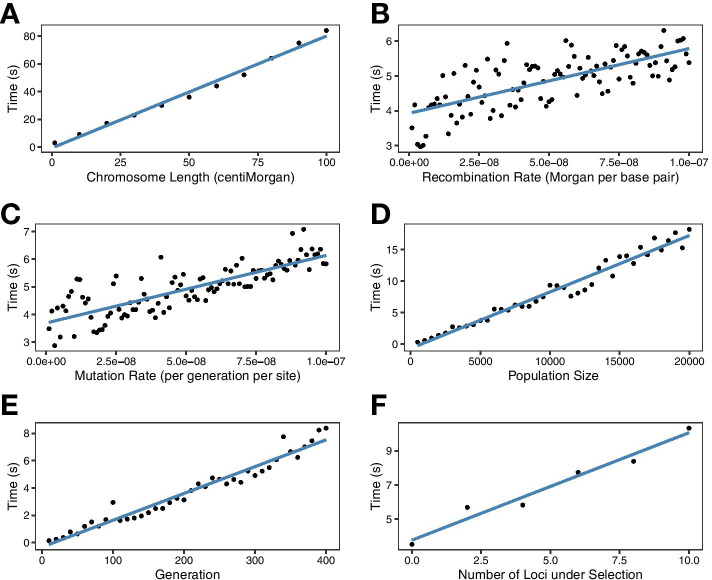
Performance evaluation of *AdmixSim 2*. **A** Varying chromosome length (centiMorgan). **B** Varying recombination rate (Morgan per base pair). **C** Varying mutation rate (per generation per site). **D** Varying population size. **E** Varying generation. **F** A varying number of loci under selection. The time cost increased linearly with the increase of the corresponding factor and was relatively low

**Fig. 6:**
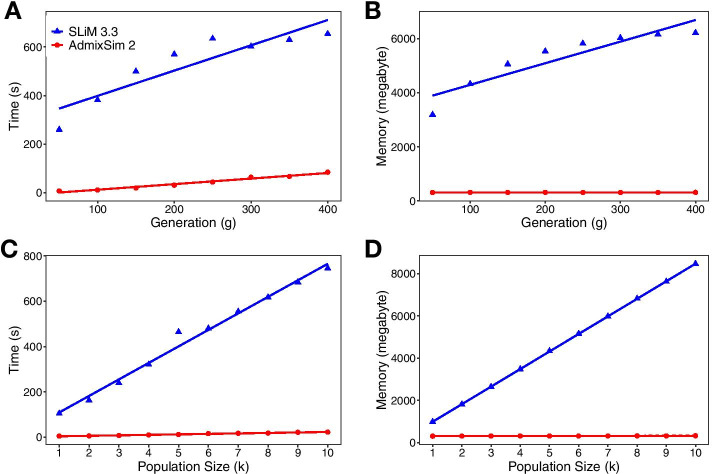
Performance comparison of *AdmixSim 2* and *SLiM 3.3*. **A** Time cost with different simulation generations. **B** Memory cost with different simulation generations. **C** Time cost with different population sizes. **D** Memory cost with different population sizes. Here the memory cost is the maximum resident set size during the simulation. Both simulators demonstrate a linear relationship with the generations or population size. The runtime and memory cost of *AdmixSim 2* is much less than *SLiM 3.3*

Further, we evaluated the time cost of *AdmixSim 2* using a HI model with equal contribution from the two ancestral populations. Six distinct factors were considered in the evaluation by varying values of one and holding the rest constant, including chromosome length, recombination rate, mutation rate, admixed population size in simulation, end generation, and a number of loci under selection (Additional file 6: Table S3). The time cost exhibited an approximately linear increase with the value of the condition rising and was relatively low (Fig. 5). Later, we compare the runtime and memory cost of *AdmixSim 2* and *SLiM 3.3* [16] using the population Yoruba in Ibadan, Nigeria (YRI) from the KGP dataset (Fig. 6). Here, for simplicity, we simulated the case that YRI evolves for a certain number of generations without gene flow from other populations. Totally 1,055,452 SNVs on chromosome 22 were used for simulation. The simulated chromosome length was about 50 Mb. The uniform recombination and mutation rate were set as 10^−8^ Morgan per base pair and 10^−8^ per generation per site. We record the runtime and peak memory usage in changing the population size (1000, 2000, 3000, 4000, 5000, 6000, 7000, 8000, 9000, 10,000) and simulated generation (50, 100, 150, 200, 250, 300, 350, 400). All tests were conducted on 96-core Intel Xeon Platinum 9242 CPU 2.30 GHz computer servers. *SLiM 3.3* was compiled under the release build. The runtime and memory cost for both simulators are approximately linear functions of generations or population size. Notably, the memory cost of *AdmixSim 2* is almost constant, while for *SLiM 3.3*, it is much higher and growing faster. These results indicate that *AdmixSim 2* is feasible and efficient in massive simulations.

## Discussion

Since *AdmixSim 2* was designed to focus on simulating the admixture process, the pre-admixture data can be generated from different strategies. In human genetics, users can use the genetic data from public data sets like the KGP, the Estonian Biocentre Human Genome Diversity Panel (EGDP), the Simons Genome Diversity Project (SGDP), and the International HapMap Project. Since *AdmixSim 2* can simulate the scenario that the ancestral populations evolve same generations as the admixed population, it is practical to use the populations from these public datasets as ancestral populations. Besides, populations from these datasets could be used as proxy populations in local ancestry inference. With the advance of genotyping and technologies, the ability to generate large amounts of sequence data in a relatively short amount of time is helping to enable a wide range of genetic analysis applications. Therefore, the pre-admixture genetic data can be more easily obtained in the future owing to advanced sequencing technologies. More generally, users can use coalescent approaches like *msprime* [26] or forward-in-time methods like *SLiM 3.3* to simulate the pre-admixture data. This approach can be applied to both human and non-human species. For instance, the documentation of *msprime* (https://tskit.dev/msprime/docs/stable/demography.html) gave the code to simulate African, European, and East Asian individuals based on the Out of Africa model developed by Gutenkunst et al. [27] from the HapMap Project data. Besides, the manual of *SLiM 3.3* (page 99) implemented a model of human evolution presented by Gravel et al. in 2011 [28] based on the KGP data. Last but not least, the output haplotype data of *AdmixSim 2* can also be used as the input of further simulations. The first three output files (haplotype data, individual information, and updated SNV information) of *AdmixSim 2* take the same format as the corresponding input, and they can be used for subsequent simulations with a new demographic model.

*AdmixSim 2* record the recombination segments rather than the whole haplotype data, which brings a proportion of efficiency for the simulation. The way of segment representation in *AdmixSim 2* is similar to that implemented in *forqs*, but there is some slight difference. Each haplotype chunk in *forqs* is represented by two numbers (position, id): the position where it begins, and the identifier of the founding haplotype from which it is derived. For *AdmixSim 2*, each segment is identified by the start physical position, the end physical position, and the ancestral label it originates from. Despite this difference is not seemly substantial, we believe specify an ending physical position is more convenient to calculate the selection fitness and extract segmental sequence data without indexing the next segment. Besides, we noticed that *forqs* is mainly used to simulate the scenarios that recombination and/or natural selection on polygenic quantitative traits within a relatively small number of generations, while the emphasis of *AdmixSim 2* is on simulating complex population admixture events more flexibly. Previous studies have demonstrated that a segment-based simulator is not as efficient as array-based simulators (like *SLiM*) when involving demographic events taking place over thousands of generations [18]. Nevertheless, we here mainly focus on the studies of modern humans, and the history usually much less than 1000 generations for typical admixed populations of modern humans.

The previous version, *AdmixSim*, has been used in many evolutionary and population genetic studies, including those exploring the genetic history of Xinjiang’s Uyghurs [23], validating statistical tool *CAMer* [29], *MultiWaver* series software [22, 30], and *Libgdrift* [31]. We summarized the similarities and differences between *AdmixSim* and *AdmixSim 2* (Table 2). We expect *AdmixSim 2* to be widely used in population admixture studies, such as modeling complicated demographic history, validating methods for local ancestry inference, and dating population admixture. In addition, autosomal and sex chromosomal data are both supported. This is particularly useful in studying sex-biased admixture.

**Table 2:** Comparisons between *AdmixSim* and *AdmixSim 2*

Features^c^	***AdmixSim***	***AdmixSim 2***
Admixture	One admixed population	Multiple^b^ admixed populations^a^
		Sex-biased
Recombination	Locus-specific/ constant	Locus-specific/constant^a^
Mutation	No	Locus-specific/constant^a^
Natural selection	No	Multiple^b^-locus^a^
		Multiple^b^ regions
		Population-specific
		Sex-specific
Chromosome type	Autosome	Autosome/X chromosome^a^

^a^Different rows in the feature descriptions can be realized simultaneously in a single simulation process. Terms in a row separated by ‘/’ are mutually exclusive

^b^All terms with ‘multiple’ support the corresponding scenarios of ‘single'

^c^The first four features are allowed to be absent

Nevertheless, there is still room for improvement in *AdmixSim 2*. As in many other programs, we here also assume non-overlapping and discrete generations, which may have some limitations in practical applications. It is possible to consider overlapping and continuous generations in future versions. In addition, de novo mutation is not considered for determining individual fitness during simulation of natural selection, and the potential effect on admixture inference is to be further evaluated. Furthermore, the tree sequence recording (TSR) algorithm [32] implemented in *SLiM 3.3* provides the necessary information to detect coalescence events and construct the genealogical tree at each chromosome position, although applying the TSR algorithm has a large impact on the performance of models, in terms of both runtime and memory usage (page 40 of *SLiM 3.3* manual). The TSR algorithm has also been applied into many other simulators like *msprime* and inference methods like *tsinfer* [33]. Nonetheless, there is no single simulation tool is ideal for all cases and some other simulation tools are suitable for a few special scenarios. For example, in the framework of selection, *SLiM 3.3*, *SELAM*, and *admix’em* consider the cases including phenotypic selection, epistatic selection, or balancing selection. Although *AdmixSim 2* does not cover the comprehensive scenarios of mate choice as some other simulators, it implements sex-biased admixture (different admixture proportions for two sexes) and sex-specific selection (different selection coefficients for two sexes).

## Conclusions

In summary, *AdmixSim 2* is an individual-based, forward-time simulator based on the extended WF model, which can be used to efficiently simulate one or more admixed populations under complex demographic scenarios. *AdmixSim 2* is highly flexible and it can implement combinations of multiple parameter settings to simulate admixture, recombination, mutation, and natural selection. Furthermore, *AdmixSim 2* can be used to simulate admixture scenarios for sex chromosomes and non-human species.

## Supplementary Information

**Additional file 1: Figure S1**. Admixture models of population (A) African America (B) Mexican and (C) Uyghur. The numbers on the arrows represent the corresponding admixture proportions. The time on the left of each model represent the admixture generations

**Additional file 2: Figure S2**. Simulation of Mexican admixture pattern. (A) PCA results. The left is the result of empirical data and the right is the result of simulated data. The patterns of two results are quite similar. (B) Segment length proportion. The proportion of each ancestry was calculated based on the sum of the corresponding segment length. The average proportion of European, African, and Native American were 0.506, 0.054, and 0.440, which is consistent with proportions set in the admixture model (European: 0.512, African: 0.052, Native American: 0.436). (C) Supervised admixture analysis results at K = 3. The left is the result of empirical data and the right is the result of simulated data. There is no marked difference between these two results. (D) Mutation number counts. The green histogram represents the simulation value and the red curve is derived from the theoretical Poisson distribution. The p-value was calculated using the chi-square goodness of fit test. The chromosome length was approximate 2.48 Morgan and the mutation rate was set as 10-8 per generation per site. As a result, after simulating 18 generations, the average mutation number of each haplotype is about 45

**Additional file 3: Figure S3**. Simulation of Uyghur admixture pattern. (A) PCA results. The left is the result of empirical data and the right is the result of simulated data. The patterns of two results are quite similar. (B) Segment length proportion. The proportion of each ancestry was calculated based on the sum of the corresponding segment length. The average proportion of East Asian, Siberian, West Eurasian, and South Asian were 0.33, 0.17, 0.36, and 0.14, which is consistent with proportions in the admixture model (East Asian: 0.35, Siberian: 0.15, West Eurasian: 0.35, South Asian: 0.15). (C) Supervised admixture analysis results at K = 4. The left is the result of empirical data and the right is the result of simulated data. There is no marked difference between these two results. (D) Mutation number counts. The green histogram represents the simulation value and the red curve is the Poisson distribution, theoretically. The p-value was calculated using the chi-square goodness of fit. The chromosome length was about 2.48 Morgan and the mutation rate was set as 10-8 per generation per site. Therefore, after simulating 150 generations, the average mutation number of each haplotype is about 372

**Additional file 4: Table S1**. Parameter settings of typical admixture pattern

**Additional file 5: Table S2**. Parameter settings of selection fixation test

**Additional file 6: Table S3**. Parameter settings of performance evaluation

## Data Availability

*AdmixSim 2*: https://www.picb.ac.cn/PGG/resource.php or https://github.com/Shuhua-Group/AdmixSim2. Operating system(s): Linux. Programming language: C++ and Python. License: GNU GPL v3.0. Any restrictions to use by non academics: None. The availability of public software and data used in the current study are listed as below: *MultiWaver* series software: https://www.picb.ac.cn/PGG/resource.php or https://github.com/Shuhua-Group/MultiWaveInfer2.0. 1000 Genomes Project data: https://www.internationalgenome.org. Human Origins Project data: https://reich.hms.harvard.edu/datasets, available through the European Nucleotide Archive under accession number PRJEB6272.

## Abbreviations

EGDP: The Estonian biocentre human genome diversity panel
GSR: Genetic simulation resources
HI: Hybrid isolation
KGP: 1000 Genomes Project
PCA: Principal component analysis
SGDP: The Simon genome diversity project
SNV: Single nucleotide variation
WF: Wright–Fisher
YRI: Yoruba in Ibadan, Nigeria
TSR: Tree sequence recording
