# Supplementary material for: AdmixSim 2: a forward-time simulator for modeling complex population admixture
Source: BMC Bioinformatics. 2021 Oct 18;22:506. doi: 10.1186/s12859-021-04415-x (PMC8522168; doi:10.1186/s12859-021-04415-x)
Supplement: Supplementary file 1 — Additional file 1: Figure S1. Admixture models of population (A) African America (B) Mexican and (C) Uyghur. The numbers on the arrows represent the corresponding admixture proportions. The time on the left of each model represent the admixture generations [file 12859_2021_4415_MOESM1_ESM.docx]

**Figure S1. Admixture models of populations** **(A) African American** **(B) Mexican** and **(C) Uyghur**. The numbers on the arrows represent the corresponding admixture proportions. The time on the left of each model represent the admixture generations.
