# Supplementary material for: AdmixSim 2: a forward-time simulator for modeling complex population admixture
Source: BMC Bioinformatics. 2021 Oct 18;22:506. doi: 10.1186/s12859-021-04415-x (PMC8522168; doi:10.1186/s12859-021-04415-x)
Supplement: Supplementary file 2 — Additional file 2: Figure S2. Simulation of Mexican admixture pattern. (A) PCA results. The left is the result of empirical data and the right is the result of simulated data. The patterns of two results are quite similar. (B) Segment length proportion. The proportion of each ancestry was calculated based on the sum of the corresponding segment length. The average proportion of European, African, and Native American were 0.506, 0.054, and 0.440, which is consistent with proportions set in the admixture model (European: 0.512, African: 0.052, Native American: 0.436). (C) Supervised admixture analysis results at K = 3. The left is the result of empirical data and the right is the result of simulated data. There is no marked difference between these two results. (D) Mutation number counts. The green histogram represents the simulation value and the red curve is derived from the theoretical Poisson distribution. The p-value was calculated using the chi-square goodness of fit test. The chromosome length was approximate 2.48 Morgan and the mutation rate was set as 10-8 per generation per site. As a result, after simulating 18 generations, the average mutation number of each haplotype is about 45 [file 12859_2021_4415_MOESM2_ESM.docx]

**Figure S2. Simulation of Mexican admixture pattern.** **(A) PCA results.** The left is the result of empirical data and the right is the result of simulated data. The patterns of two results are quite similar. **(B) Segment length proportion.** The proportion of each ancestry was calculated based on the sum of the corresponding segment length. The average proportion of European, African, and Native American were 0.506, 0.054, and 0.440, which is consistent with proportions set in the admixture model (European: 0.512, African: 0.052, Native American: 0.436). **(C) Supervised admixture analysis results at K = 3.** The left is the result of empirical data and the right is the result of simulated data. There is no marked difference between these two results. **(D) Mutation number counts.** The green histogram represents the simulation value and the red curve is derived from the theoretical Poisson distribution. The *p*-value was calculated using the chi-square goodness of fit test. The chromosome length was approximate 2.48 Morgan and the mutation rate was set as 10^-8^ per generation per site. As a result, after simulating 18 generations, the average mutation number of each haplotype is about 45.
