# Supplementary material for: AdmixSim 2: a forward-time simulator for modeling complex population admixture
Source: BMC Bioinformatics. 2021 Oct 18;22:506. doi: 10.1186/s12859-021-04415-x (PMC8522168; doi:10.1186/s12859-021-04415-x)
Supplement: Supplementary file 3 — Additional file 3: Figure S3. Simulation of Uyghur admixture pattern. (A) PCA results. The left is the result of empirical data and the right is the result of simulated data. The patterns of two results are quite similar. (B) Segment length proportion. The proportion of each ancestry was calculated based on the sum of the corresponding segment length. The average proportion of East Asian, Siberian, West Eurasian, and South Asian were 0.33, 0.17, 0.36, and 0.14, which is consistent with proportions in the admixture model (East Asian: 0.35, Siberian: 0.15, West Eurasian: 0.35, South Asian: 0.15). (C) Supervised admixture analysis results at K = 4. The left is the result of empirical data and the right is the result of simulated data. There is no marked difference between these two results. (D) Mutation number counts. The green histogram represents the simulation value and the red curve is the Poisson distribution, theoretically. The p-value was calculated using the chi-square goodness of fit. The chromosome length was about 2.48 Morgan and the mutation rate was set as 10-8 per generation per site. Therefore, after simulating 150 generations, the average mutation number of each haplotype is about 372 [file 12859_2021_4415_MOESM3_ESM.docx]

**Figure S3. Simulation of Uyghur admixture pattern.** **(A) PCA results.** The left is the result of empirical data and the right is the result of simulated data. The patterns of two results are quite similar. **(B) Segment length proportion.** The proportion of each ancestry was calculated based on the sum of the corresponding segment length. The average proportion of East Asian, Siberian, West Eurasian, and South Asian were 0.33, 0.17, 0.36, and 0.14, which is consistent with proportions in the admixture model (East Asian: 0.35, Siberian: 0.15, West Eurasian: 0.35, South Asian: 0.15). **(C) Supervised admixture analysis results at K = 4.** The left is the result of empirical data and the right is the result of simulated data. There is no marked difference between these two results. **(D) Mutation number counts.** The green histogram represents the simulation value and the red curve is the Poisson distribution, theoretically. The *p*-value was calculated using the chi-square goodness of fit. The chromosome length was about 2.48 Morgan and the mutation rate was set as 10^-8^ per generation per site. Therefore, after simulating 150 generations, the average mutation number of each haplotype is about 372.
