# Supplementary material for: AdmixSim 2: a forward-time simulator for modeling complex population admixture
Source: BMC Bioinformatics. 2021 Oct 18;22:506. doi: 10.1186/s12859-021-04415-x (PMC8522168; doi:10.1186/s12859-021-04415-x)
Supplement: Supplementary file 4 — Additional file 4: Table S1. Parameter settings of typical admixture pattern [file 12859_2021_4415_MOESM4_ESM.docx]

**Table S1. Parameter settings of typical admixture pattern**

| Admixture pattern | Data Source | SNV counts | Ancestral populations | Ancestral population size |
| --- | --- | --- | --- | --- |
| African American | 1000 Genome Project | 6,196,135 | European, African^a^ | 50 |
| Mexican | Human Origin | 50,824 | European, African^b^, Native American^c^ | 20 |
| Uyghur | Human Origin | 46,433 | East Asian, Siberian, West Eurasian, South Asian^d^ | 10 |

^a^ Use Utah residents with Northern and Western European ancestry (CEU) and Yoruba in Ibadan, Nigeria (YRI) as representative population of European and African for admixture pattern of African American.

^b^ Use French and Yoruba as representative population of European and African for admixture pattern of Mexican.

^c^ Use Cabecar and Pima as representative populations of Native American.

^d^ Use Han, Chukchi, Sardinian, and Mala as representative populations of East Asian, Siberian, West Eurasian, and South Asian.
