# Supplementary material for: AdmixSim 2: a forward-time simulator for modeling complex population admixture
Source: BMC Bioinformatics. 2021 Oct 18;22:506. doi: 10.1186/s12859-021-04415-x (PMC8522168; doi:10.1186/s12859-021-04415-x)
Supplement: Supplementary file 5 — Additional file 5: Table S2. Parameter settings of selection fixation test [file 12859_2021_4415_MOESM5_ESM.docx]

**Table S2. Parameter settings of selection fixation test**

| Chromosome length | Admixture model | Admixture proportion | Ancestral population size | Initial frequency | Selection coefficients | Repeat times |
| --- | --- | --- | --- | --- | --- | --- |
| 1 centiMorgan | HI model | 1:1 | 100 | 5%, 10%, 15%, 20% | (0.01, 0.1; 0.01) | 100 |
