# Supplementary material for: AdmixSim 2: a forward-time simulator for modeling complex population admixture
Source: BMC Bioinformatics. 2021 Oct 18;22:506. doi: 10.1186/s12859-021-04415-x (PMC8522168; doi:10.1186/s12859-021-04415-x)
Supplement: Supplementary file 6 — Additional file 6: Table S3. Parameter settings of performance evaluation [file 12859_2021_4415_MOESM6_ESM.docx]

**Table S3. Parameter settings of performance evaluation**

| Parameter | Fixed value | Varying value |
| --- | --- | --- |
| chromosome length (centiMorgan) ^a^ | 1 | 1, (10, 100; 10) |
| recombination rate (Morgan per base pair) | 10^-8^ | (10^-9^, 10^-7^; 10^-9^) |
| mutation rate (per generation per site) | 10^-8^ | (10^-9^, 10^-7^; 10^-9^) |
| population size | 5000 | (500, 20000; 500) |
| generation | 200 | (10, 400; 10) |
| number of loci under selection | 0 | (0, 10; 2) |

^a^ Every $4\times{10}^{-5}$ centiMorgan one locus.
